# Supplementary material for: Inverse Design of Whispering-Gallery Nanolasers with Tailored Beam Shape and Polarization
Source: ACS Photonics. 2022 Nov 30;10(4):968–76. doi: 10.1021/acsphotonics.2c01165 (PMC10119977; doi:10.1021/acsphotonics.2c01165)
Supplement: Supplementary file 1 — ph2c01165_si_001.pdf [file ph2c01165_si_001.pdf]

# Supporting Information: Inverse Design of Whispering-Gallery Nanolasers with Tailored Beam Shape and Polarization

Iago Diez,*,†,‡ Andrey Krysa,¶ and Isaac J. Luxmoore*,†

†Department of Engineering, University of Exeter, EX4 4QF, Exeter, United Kingdom

‡Department of Physics and Astronomy, University of Exeter, EX4 4QL, Exeter, United Kingdom

¶EPSRC National Epitaxy Facility, University of Sheffield, S1 3JD, Sheffield, United Kingdom

E-mail: ir274@exeter.ac.uk; i.j.luxmoore@exeter.ac.uk

## S1. Spatial Filtering in the Topology Optimization

The density value at the element $j$ is a weighted average of the density values of surrounding elements $i$:

$$\tilde{\rho}_j(\rho_i) = \frac{\sum_i w_{ji}\rho_i}{\sum_i w_{ji}} \tag{1}$$

where the weighting function $w_{ji}$ is given by the linearly decaying function which depends on the distance $d_{ji}$ between elements $i$ and $j$ and the blur radius $R_b$:

$$w_{ji} = R_b - d_{ji} \quad \text{for} \quad d_{ji} \leq R_b \qquad , \qquad w_{ji} = 0 \quad \text{for} \quad d_{ji} > R_b$$

$$\text{with} \qquad d_{ji} = \sqrt{\left(x_i - x_j\right)^2 + \left(y_i - y_j\right)^2}$$

We chose $R_b = 20$ nm, which is within the capabilities of electron beam lithography.

## S2. Adjoint Calculation of the Gradient

This section covers the key concepts of the adjoint method to obtain the gradient of the FoM with respect to the design variables. The following mathematical development is similar to Refs.[1–4]

The nanophotonics optimisation aim is to find a device geometry, given by the spatial distribution of a dielectric within a region $\xi$, that maximises an analytical FoM function $\mathcal{F}(\mathbf{E})$ over a region $\Omega$ (Figure S1(a)). In general, $\mathcal{F}$ can be a function of both the electric and magnetic field. However,here we will restrict the discussion to the case of only depending on the electric field for simplicity and because this is the case for the FoM used in this work.

$$\mathcal{F} = \left| \int_\Omega \mathbf{E}_m^* \cdot \mathbf{E} \, d\mathbf{x} \right| \equiv |f| \tag{2}$$

where $\Omega$ is the FoM plane over which the target field is desired, $\mathbf{E}_m$ is the target electric field, and both $\mathbf{E}$ and $\mathbf{E}_m$ are normalised by $\sqrt{\int_\Omega |\mathbf{E}|^2 \, d\mathbf{x}}$ and $\sqrt{\int_\Omega |\mathbf{E}_m|^2 \, d\mathbf{x}}$, respectively, and $f$ refers to the integral of the complex projection. Notice that $\mathcal{F}$ is real whereas $f$ can be complex in general. The variation in the FoM can be expressed as:

$$\delta\mathcal{F} = \frac{\partial \mathcal{F}}{\partial f}\delta f + \frac{\partial \mathcal{F}}{\partial f^*}\delta f^* = 2\mathrm{Re}\left\{ \frac{\partial \mathcal{F}}{\partial f}\delta f \right\} = 2\mathrm{Re}\left\{ \frac{f^*}{2|f|}\delta f \right\} = \mathrm{Re}\left\{ e^{-i\arg(f)}\delta f \right\} \tag{3}$$

where the following property of complex numbers was used: if $z$ is a complex number $\rightarrow$ $z + z^* = 2\,\mathrm{Re}\{z\}$, and also that $\mathcal{F} = (ff^*)^{1/2}$.

The optimisation algorithm is derived from analysing how a small geometrical variation, which is linked to a small variation in the electric field, $\delta\mathbf{E}$, causes a small variation in the

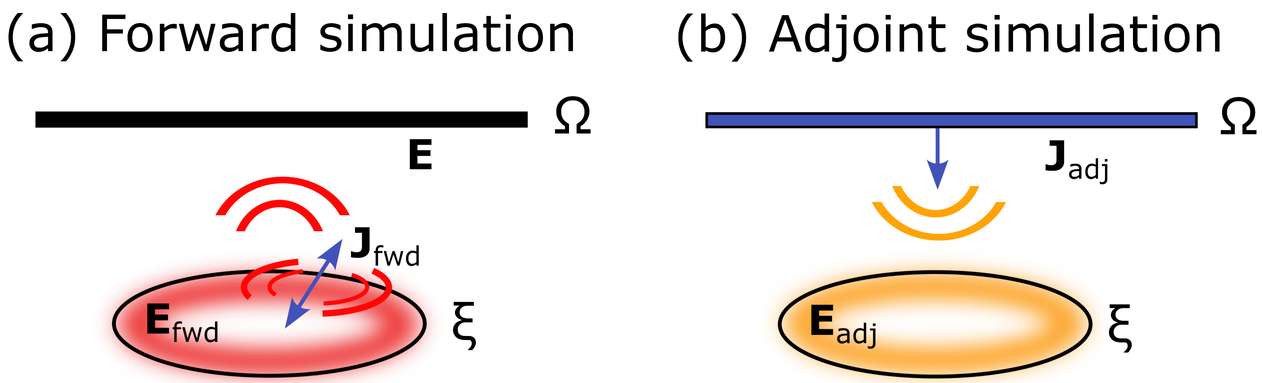


Figure S1: Schematic of the configuration of sources and recorded fields for (a) the forward simulation and (b) the adjoint simulation. In (a) the source $\mathbf{J}_{fwd}$ is the electric dipole placed inside the disc, $\mathbf{E}_{fwd}$ is the electric field at the design region $\xi$ and $\mathbf{E}$ is the electric field at the plane $\Omega$ where the target mode is desired. In (b) the source $\mathbf{J}_{adj}$ is the conjugate of the desired electric field at $\Omega$ and $\mathbf{E}_{adj}$ is the electric field at the design region $\xi$.

FoM, and thus in $f$ which can be expressed as:

$$\delta f = \int_{\Omega} \mathbf{E}_m^* \cdot \delta \mathbf{E} \, d\mathbf{x} \tag{4}$$

The variations in the electric field $\delta\mathbf{E}$ at a voxel $\mathbf{x} \in \Omega$ stem from the electric dipoles arisen from the induced polarisation density, $\mathbf{P}_{ind}$, at $\mathbf{x}' \in \xi$ upon geometrical changes, i.e. due to changes in the dielectric permittivity at $\mathbf{x}'$. Green's functions enables the connection between oscillating dipole current source and their produced fields: $\mathcal{G}(\mathbf{x}, \mathbf{x}')$ is the dyadic Green's function that represents the electric field at $\mathbf{x}$ produced by a point dipole current oscillating at $\mathbf{x}'$. With this, the variation in the electric field can be expressed as a function of the polarisation density:[5]

$$\delta \mathbf{E}\left(\mathbf{x}\right) = \mu_0 \omega^2 \int_{\xi} \mathcal{G}(\mathbf{x}, \mathbf{x}') \, \mathbf{P}_{ind}(\mathbf{x}') \, d\mathbf{x}' \tag{5}$$

Thus, the variation in the complex projection can be rewritten as:

$$\delta f = \mu_0 \omega^2 \int_{\Omega} d\mathbf{x} \int_{\xi} d\mathbf{x}' \; \mathbf{E}_m^*(\mathbf{x}) \, \mathcal{G}(\mathbf{x}, \mathbf{x}') \, \mathbf{P}_{ind}(\mathbf{x}') \tag{6}$$

Due to Lorenz reciprocity, the relationship between a current source (oscillating dipole) and the resulting field is unchanged if the source position and the point where the field is measured are interchanged.[6] This symmetry argument for electric dipoles is expressed with Green's functions as: $\mathcal{G}(\mathbf{x}, \mathbf{x}') = \mathcal{G}(\mathbf{x}', \mathbf{x})^T$. Thus, the integrands of the previous equation can be rearranged:

$$\delta f = \int_{\xi} d\mathbf{x}' \, \mathbf{P}_{ind}(\mathbf{x}') \, \mu_0 \omega^2 \int_{\Omega} d\mathbf{x} \, \mathcal{G}(\mathbf{x}', \mathbf{x})^T \, \mathbf{E}_m^*(\mathbf{x}) \tag{7}$$

The term $\mathcal{G}(\mathbf{x}', \mathbf{x})^T \, \mathbf{E}_m^*(\mathbf{x})$ represents the electric field at $\mathbf{x}'$ produced by an electric dipole at $\mathbf{x}$ with amplitude $\mathbf{E}_m^*(\mathbf{x})$. Therefore the integration over the region $\Omega$ represents the electric field produced at $\mathbf{x}'$ by all electric dipoles placed throughout $\Omega$. This field is known as the *adjoint field* $\mathbf{E}_{adj}$, and the source of amplitude $\mathbf{E}_m^*(\mathbf{x})$ producing the field is known as the *adjoint source* (Figure S1):

$$\mathbf{E}_{adj}(\mathbf{x}') \equiv \mu_0 \omega^2 \int_{\Omega} d\mathbf{x} \, \mathcal{G}(\mathbf{x}', \mathbf{x})^T \, \mathbf{E}_m^*(\mathbf{x}) \tag{8}$$

The variation in $f$ can now be simplified to:

$$\delta f = \int_{\xi} \mathbf{P}_{ind}(\mathbf{x}') \cdot \mathbf{E}_{adj}(\mathbf{x}') d\mathbf{x}' \tag{9}$$

The type of geometrical modification considered here is a change in permittivity within an infinitesimal voxel located at any given point $\mathbf{x}_j \in \xi$. The induced polarisation arisen from modifying the permittivity by an amount $\delta\varepsilon$ can be expressed as:[7] $\mathbf{P}_{ind}(\mathbf{x}') = \varepsilon_0 \, \delta\varepsilon \, \mathbf{E}_{fwd}(\mathbf{x}')$, where $\mathbf{E}_{fwd}$ is the electric field obtained from the forward simulation throughout the design region $\xi$. The variation in $f$ due to this new permittivity variation within the voxel of volume $V_j$ is then:

$$\delta f = \varepsilon_0 \, \delta\varepsilon \int_{V_j} \mathbf{E}_{fwd}(\mathbf{x}') \cdot \mathbf{E}_{adj}(\mathbf{x}') d\mathbf{x}' \tag{10}$$

As the volume of the voxel is considered infinitesimally small, the integral can be reduced

to the values of the electric field at the voxel centre $\mathbf{x}'_j$.

$$\delta f_j = V_j \varepsilon_0 \delta\varepsilon_j \left[\mathbf{E}_{fwd}(\mathbf{x}'_j) \cdot \mathbf{E}_{adj}(\mathbf{x}'_j)\right] \tag{11}$$

If we introduce this expression of $\delta f$ into Equation 3, the variation of $\mathcal{F}$ due to the dielectric change at $\mathbf{x}'_j$ is:

$$\delta\mathcal{F}_j = \delta\varepsilon_j V_j \varepsilon_0 \,\mathrm{Re}\Big\{e^{-i\arg(f)} \left[\mathbf{E}_{fwd}(\mathbf{x}'_j) \cdot \mathbf{E}_{adj}(\mathbf{x}'_j)\right]\Big\} \tag{12}$$

We define the gradient $g_j$ of the FoM function with respect to the dielectric permittivity of a voxel $j \in \xi$ as:

$$g_j \equiv \frac{\delta\mathcal{F}_j}{\delta\varepsilon_j} = \frac{\delta\mathcal{F}}{\delta\varepsilon}(\mathbf{x}'_j) = V_j \varepsilon_0 \,\mathrm{Re}\Big\{e^{-i\arg(f)} \left[\mathbf{E}_{fwd}(\mathbf{x}'_j) \cdot \mathbf{E}_{adj}(\mathbf{x}'_j)\right]\Big\} \tag{13}$$

If for each voxel $j$ we select $\delta\varepsilon_j = |a|\, g_j$, for any constant $a$, this will make $\delta\mathcal{F}_j = |a| g_j^2 > 0 \;\forall\; j$, hence updating the permittivity distribution at each voxel $j$ throughout the design region in the direction that maximises the FoM. Therefore one simple update algorithm of the dielectric permittivity $\varepsilon^{(k)}$ at iteration $k$ for any voxel $j$ could be:

$$\varepsilon_j^{(k)} = \varepsilon_j^{(k-1)} + \gamma \,\frac{g_j}{\max|g|} \tag{14}$$

where $\gamma$ is a hyperparameter that controls the evolution rate, but taking into consideration that the new permittivity value is bounded: $\varepsilon_j^{(k)} \in [\varepsilon_{\text{air}},\, \varepsilon_{\text{QW}}]$. This approach is known as *gradient steepest ascent* method. As $g_j$ is normalised by the maximum, the prefactors $V_j \varepsilon_0$ can be omitted in the definition of the gradient, for practicality.

$$g_j = \mathrm{Re}\Big\{e^{-i\arg(f)} \left[\mathbf{E}_{fwd}(\mathbf{x}'_j) \cdot \mathbf{E}_{adj}(\mathbf{x}'_j)\right]\Big\} = |D_j| \cos\left(\arg D_j - \arg f\right) \tag{15}$$

where the notation of the dot product was simplified to $D_j \equiv \mathbf{E}_{fwd}(\mathbf{x}'_j) \cdot \mathbf{E}_{adj}(\mathbf{x}'_j)$.

This gradient indicates whether the dielectric permittivity of each voxel $j$ within the design region should be increased ($g_j > 0$) or decreased ($g_j < 0$), in order to maximise $\mathcal{F}$. The evolution of the density matrix is dependent not only on the sign of the gradient $g_j$ but also on its modulus. In the case of using the density-parametrisation, the chain rule is applied for finding the gradient with respect to $\rho$: $(\delta\mathcal{F}/\delta\rho_j) = (\delta\mathcal{F}/\delta\varepsilon_j)\,(\delta\varepsilon_j/\delta\rho_j) = g_j \cdot (\varepsilon_{\mathrm{QW}} - \varepsilon_{\mathrm{air}})$. The gradient is the same except for a constant that can be merged into $\gamma$.

Although Equation 15 is the definition of the gradient, the nanolasers presented in this work were optimised with the following expression, $G$:

$$G_j = \mathrm{Re}\left\{\mathbf{E}_{fwd}(\mathbf{x}'_j) \cdot \mathbf{E}_{adj}(\mathbf{x}'_j)\right\} = |D_j| \cos\left(\arg D_j\right) \tag{16}$$

The difference between $g$ and $G$ is an overall phase: $\arg f = \arg\left\{\int_\Omega \mathbf{E}^*_m \cdot \mathbf{E}\; d\mathbf{x}\right\}$. The role of this phase in the optimisation is to account for the possible phase mismatch at $\Omega$ between the electric field of the target mode, $\mathbf{E}_m$, and the electric field $\mathbf{E}$ that is being optimised. Optimising with $g$ will produce a cavity design that generates the field at the same phase of the oscillation as $\mathbf{E}_m$. By removing this phase factor from the gradient expression, the optimisation will yield a cavity that generates the field in any phase of the oscillation but still with the same distribution in intensity, phase, and polarization as the target. This is equivalent to removing this constraint in the phase, by using Equation 16 for the expression of the gradient.

In summary, the permittivity distribution, and by extension the geometry, of the design region $\xi$ can be optimized by computing the gradient obtained from performing two electromagnetic simulations. A first simulation for calculating the forward field, and a second simulation for computing the adjoint field (Figure S1(b)).

# S3. Evolution of the Binarization Degree

The binarization degree ($B$) of the density distribution measures what fraction of the design region is close to be binary. $B$ is bounded between 0, i.e. the density of all elements is equal to 0.5; and 1, the density for all elements is either 1 or 0, i.e. binary.[8,9]

$$B = \frac{1}{0.5N} \sum_{j \in \xi}^{N} |\rho_j - 0.5| \tag{17}$$

where $\xi$ represents the design region, $\rho_j$ is the density parameter at the element $j$ and $N$ is the total number of elements in the design region.

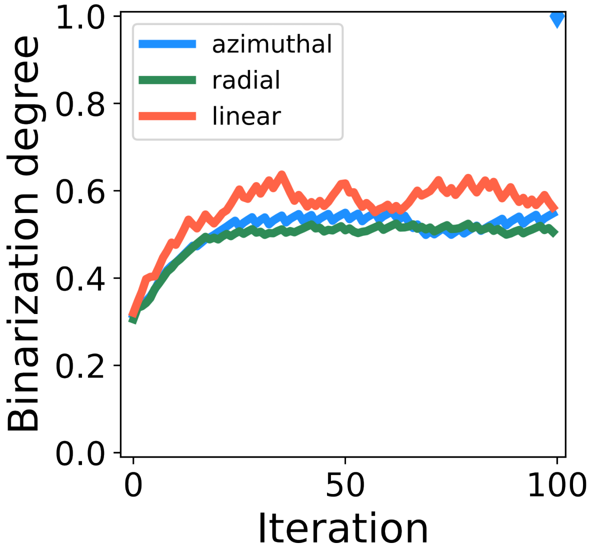

Figure S2: Evolution of the binarization degree.

## S4. Evolution of the Figure of Merit in the Far-field

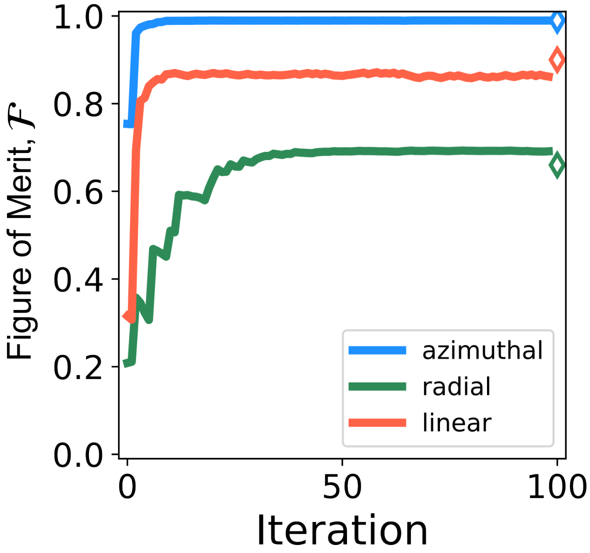


Figure S3: Figure of Merit calculated by projecting the near-field into the far-field.

## S5. Polarimetry Analysis

The Stokes parameters are obtained from six measurements of the far-field intensity at different relative orientations between a quarter-wave plate and a linear polarizer. Four of the measurements are taken by rotating the transmission axis of the linear polarizer at different angles with respect to the horizontal at 0°: $I_0$ horizontally polarized light (0°), $I_{90}$ vertically polarized light (90°), $I_{45}$ diagonally polarized light (45°), $I_{135}$ antidiagonally polarized light (135°). For the remaining two measurements a quarter-wave plate is added with its fast axis aligned at 0°: $I_{0,45}$ clockwise circularly polarized light (0°, 45°) and $I_{0,135}$ anticlockwise circularly polarized light (0°, 135°).

The Stokes parameters are calculated as:

$$S_0 = I_0 + I_{90}$$

$$S_1 = I_0 - I_{90} \quad \rightarrow \quad s_1 = S_1/S_0$$

$$S_2 = I_{45} - I_{135} \quad \rightarrow \quad s_2 = S_2/S_0$$

$$S_3 = I_{0,45} - I_{0,135} \quad \rightarrow \quad s_3 = S_3/S_0$$

The degree of polarization (DP), and degree of linear (DLP) and circular (DCP) polarization are obtained as:

$$DP = \sqrt{(s_1)^2 + (s_2)^2 + (s_3)^2}$$

$$DLP = \sqrt{(s_1)^2 + (s_2)^2}$$

$$DCP = |s_3|$$

The angle of the polarization ellipse is obtained by:

$$\Psi = \frac{1}{2}\arctan(s_2/s_1), \qquad \Psi \,\epsilon\, [-\pi/2, \pi/2]$$

The polarization of the far-field can be represented in terms of intensity and polarization, not phase, as:

$$[P_x,\ P_y] = \left[\sqrt{I_0}\cos(\Psi),\ \sqrt{I_{90}}\sin(\Psi)\right]$$

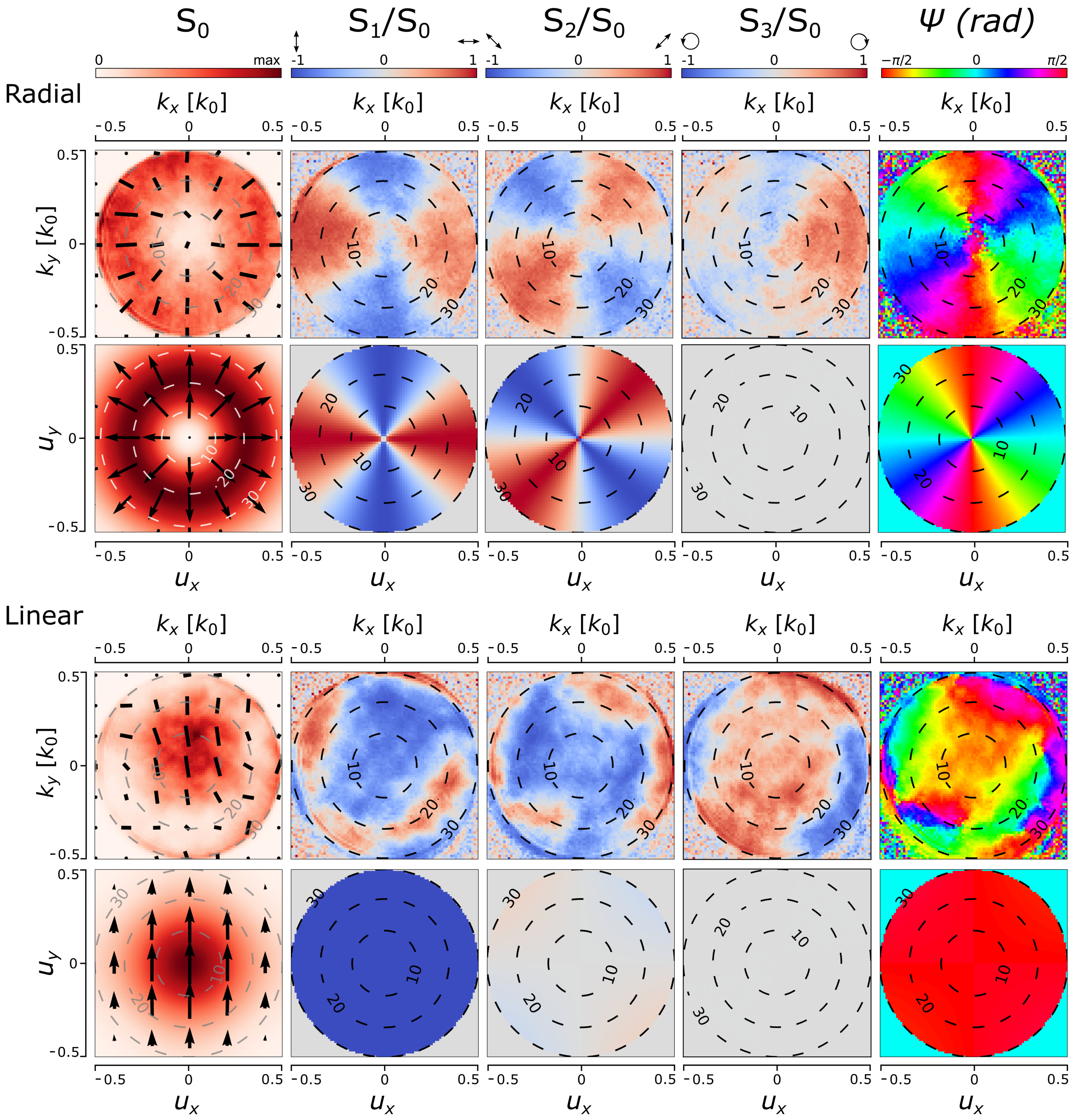


Figure S4: Stokes parameters and polarization ellipse angle $\Psi$ for the nanolasers shown in Figure 5(a) in the main text.

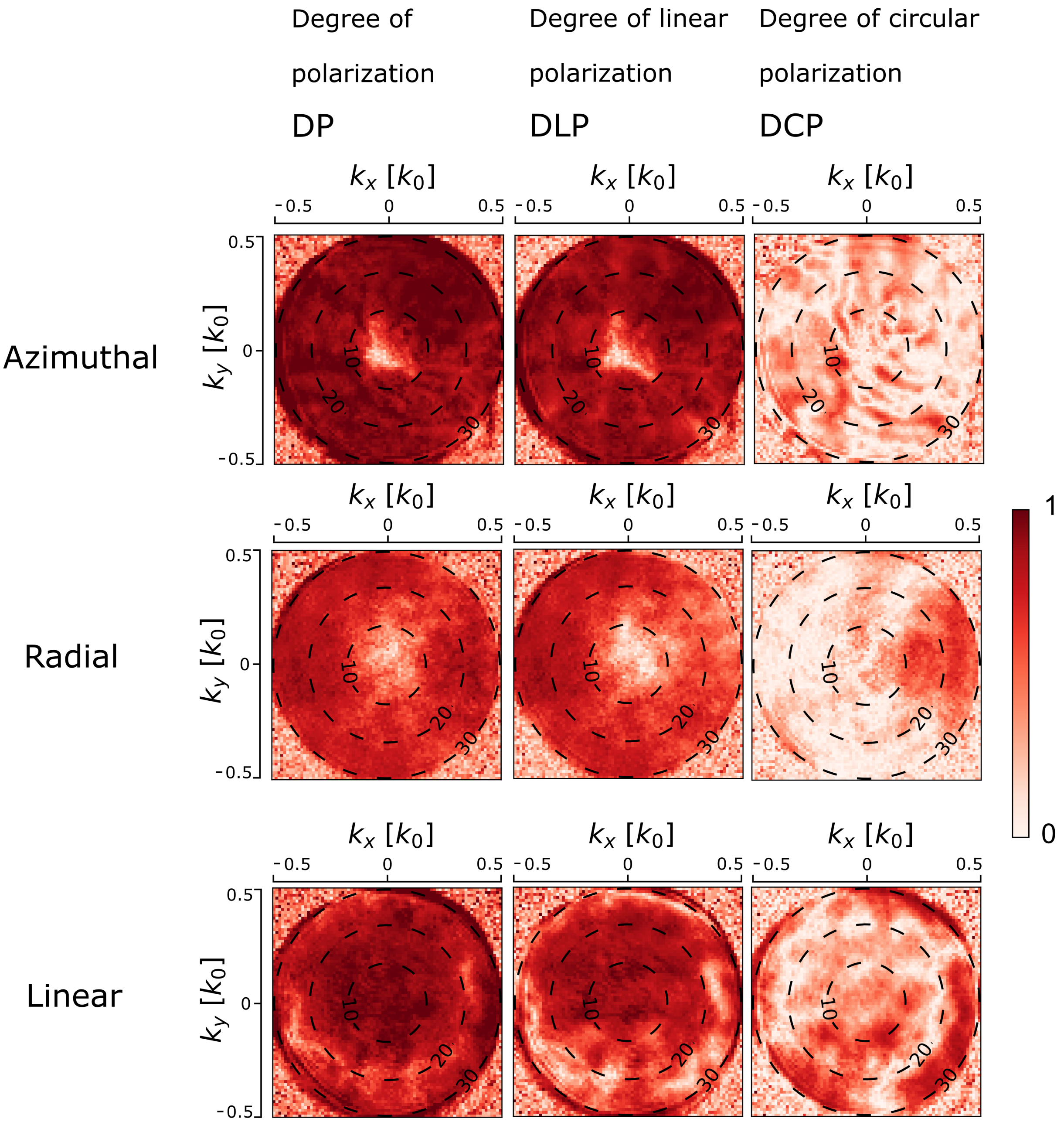


Figure S5: Degree of polarization, degree of linear and circular polarization for the nanolasers shown in Figure 4 and 5(a) and 5(b) in the main text.

# S6. Stokes Parameters of the Far-field for the Binarized Designs

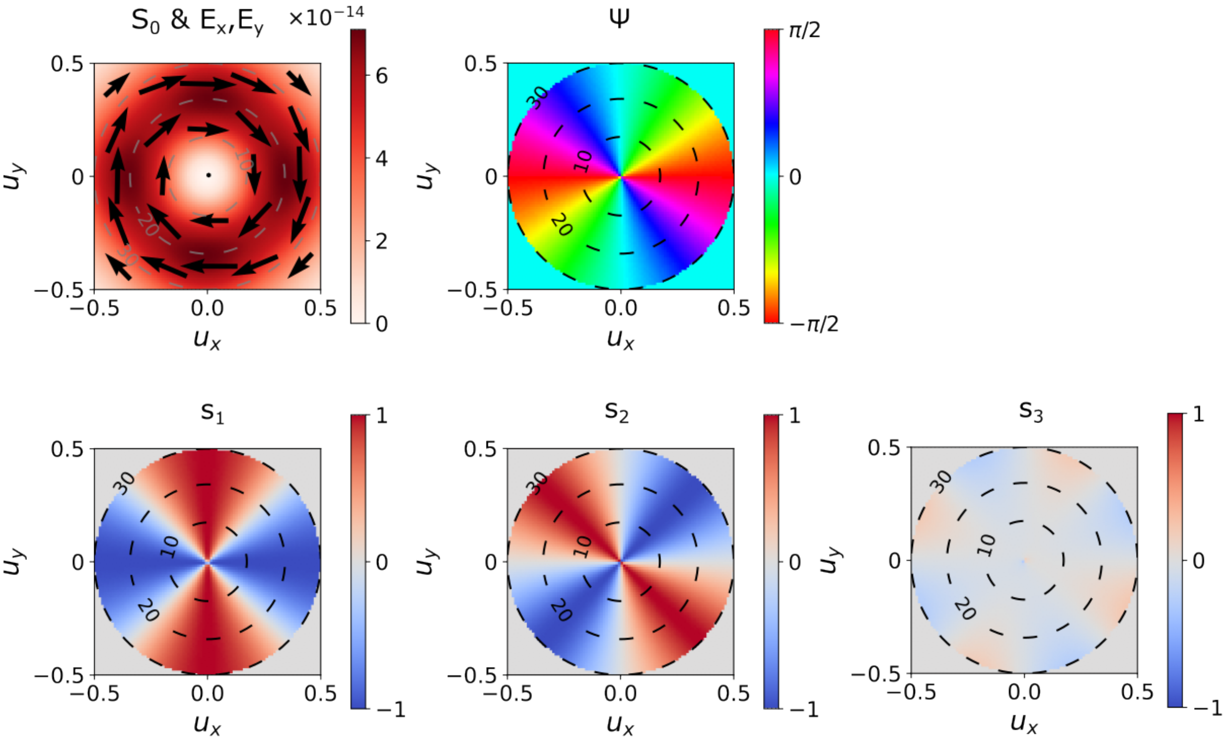


Figure S6: Stokes parameters of the far-field emitted from the AP cavity.

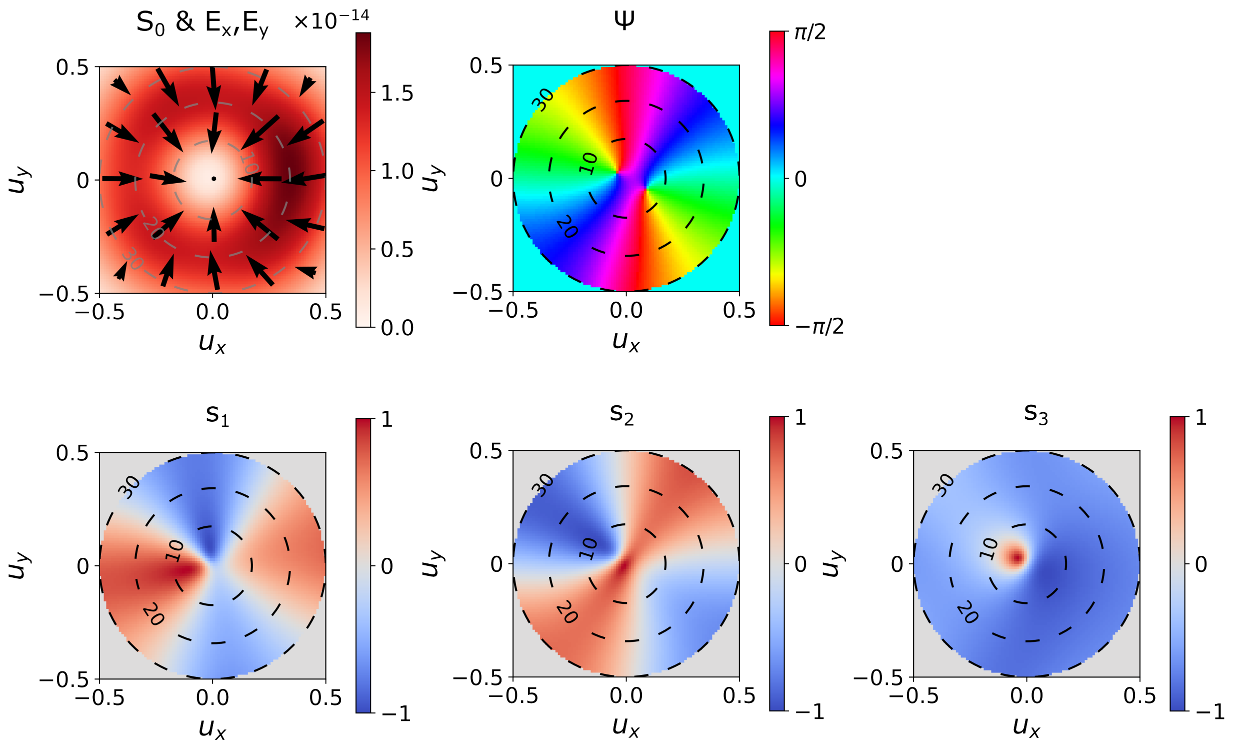


Figure S7: Stokes parameters of the far-field emitted from the RP cavity.

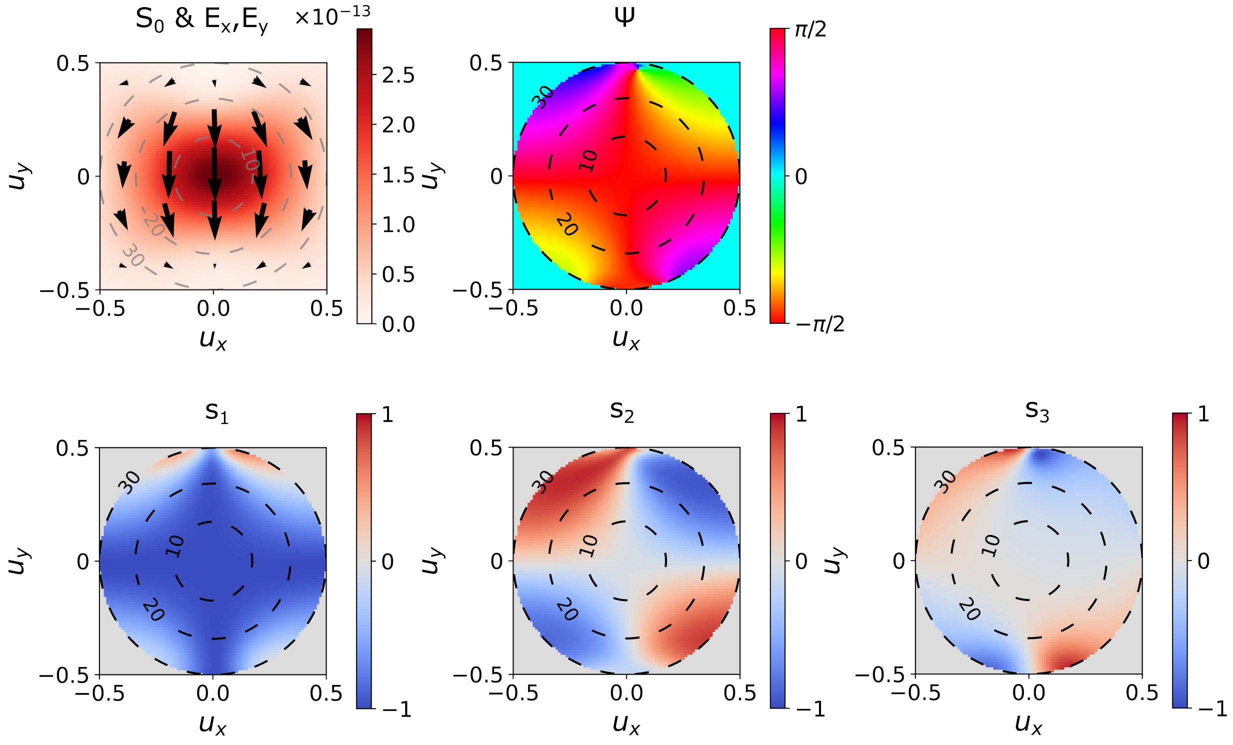


Figure S8: Stokes parameters of the far-field emitted from the LP cavity.

# S7. Overlap between Experimental and Target Far-fields

Their similarity was computed by overlapping the experimental far-field $[P_x,\ P_y]$ with the desired one $[E_{m,x},\ E_{m,y}]$. Notice that $\Psi$ allow us to recreate the polarization of the experimental field at each point of space but we do not have information of the phase of the field. That is why the modulus is done at the dot product per individual component.

$$\begin{aligned}\text{Exp. overlap} &= \frac{\sum\left(|P_x\, E_{m,x}| + |P_y\, E_{m,y}|\Delta x \Delta y\right)}{\sqrt{\sum S_0 \Delta x \Delta y}\sqrt{\sum\left(|E_{m,x}|^2 + |E_{m,y}|^2 \Delta x \Delta y\right)}} = \\ &= \frac{\sum |P_x\, E_{m,x}| + |P_y\, E_{m,y}|}{\sqrt{\sum S_0}\sqrt{\sum\left(|E_{m,x}|^2 + |E_{m,y}|^2\right)}}\end{aligned} \tag{18}$$

The mathematical description used for the target beams used in the experimental overlap were $[E_{m,x}, E_{m,y}]$ :

$$\text{azimuthal} \quad \rightarrow \quad \mathbf{E}_{AP} = -\,\mathrm{HG}_{01}\,\hat{\mathbf{x}} + \,\mathrm{HG}_{10}\,\hat{\mathbf{y}}$$

$$\text{radial} \quad \rightarrow \quad \mathbf{E}_{RP} = +\,\mathrm{HG}_{10}\,\hat{\mathbf{x}} - \,\mathrm{HG}_{01}\,\hat{\mathbf{y}}$$

$$\text{linear} \quad \rightarrow \quad \mathbf{E}_{LP} = \mathrm{G}_{00}\,\hat{\mathbf{e}}$$

where $\hat{\mathbf{x}}$ and $\hat{\mathbf{y}}$ represent the unitary vectors along the X- and Y-axis, respectively; $\hat{\mathbf{e}}$ represents an arbitrary unitary vector in the X-Y plane and $\mathrm{HG}_{pq}$ refers to the Hermite-Gaussian mode of indices $p$ and $q$ calculated from the $n$-th degree Hermite polynomial $H_n$:

$$\mathrm{G}_{00} = \exp\left(-\frac{k_x^2 + k_y^2}{w_0^2}\right)$$

$$\mathrm{HG}_{10} = \mathrm{G}_{00}\ \mathrm{H}_1\left(\sqrt{2}\frac{k_x}{w_0}\right)\ \mathrm{H}_0\left(\sqrt{2}\frac{k_y}{w_0}\right)$$

$$\mathrm{HG}_{01} = \mathrm{G}_{00}\ \mathrm{H}_0\left(\sqrt{2}\frac{k_x}{w_0}\right)\ \mathrm{H}_1\left(\sqrt{2}\frac{k_y}{w}\right)$$

with $w_0 = 0.5\, k_0$ being the beam waist in $k-$space $(k_x, k_y)$.

# S8. Characterization of the Measured Nanolasers

For each targeted radiation mode we fabricated and tested two sets of three arrays of nanolasers, each array being composed by the designs targeting one of the desired beams. Each nanolaser within these arrays had a slightly different geometry to account for the possible variability arising from the nanofabrication process and possible mismatch of the refractive index value used to simulate the QW material. The different designs were obtained by varying two parameters: the threshold $\eta$ used for binarizing the optimized gray density distribution and the scaling in size of the resulting binarized design. When compared to the optimized design, a larger (smaller) $\eta$ accounts for an over-etched (under-etched) structure. An up-scale (down-scale) in the lateral size of the geometry red-shifts (blue-shifts) the WGM wavelength. The caveat here is that the two-dimensional designs is scaled only in two spatial directions but not along their thickness direction of the nanodisc. So apart from shifting the wavelength, the WGM and its associated radiation mode might be altered too as a consequence. $\eta$ was varied in steps of 0.05 and the scale in size was varied in steps of $\times 0.01$ compared to the optimized design, which was assumed to correspond to $\eta = 0.5$ and scaling $\times 1$. Therefore the nanolasers whose far-field was analyzed were selected within the $\eta$-range of 0.45 – 0.55 and scale-range of $\times 0.90$ – $\times 1.10$. The nanolasers that exhibited best exp. overlap were the ones at $\eta = 0.5$ and scale $\times 0.99$ – 1.01 (AP), $\times 0.96$ – 0.99 (RP), $\times 1.03$ – 1.06

Out of all nanolasers measured, an ensemble of 5 to 7 nanolasers of each type was selected for the L–L curve analysis corresponding to $\eta = \{0.4, 0.45, 0.5, 0.55\}$ and a scaling in size of $\times 0.99$ – $\times 1.02$ (AP), $\times 0.96$ – $\times 0.99$ (RP), $\times 1.01$ – $\times 1.06$ (LP).

- Statistical sample size = 6 (AP), 5 (RP), 7 (LP), 4 (Conv).
- Interval of lasing wavelength: 681.63 nm – 695.13 nm (AP), 655.45 nm – 668.36 nm

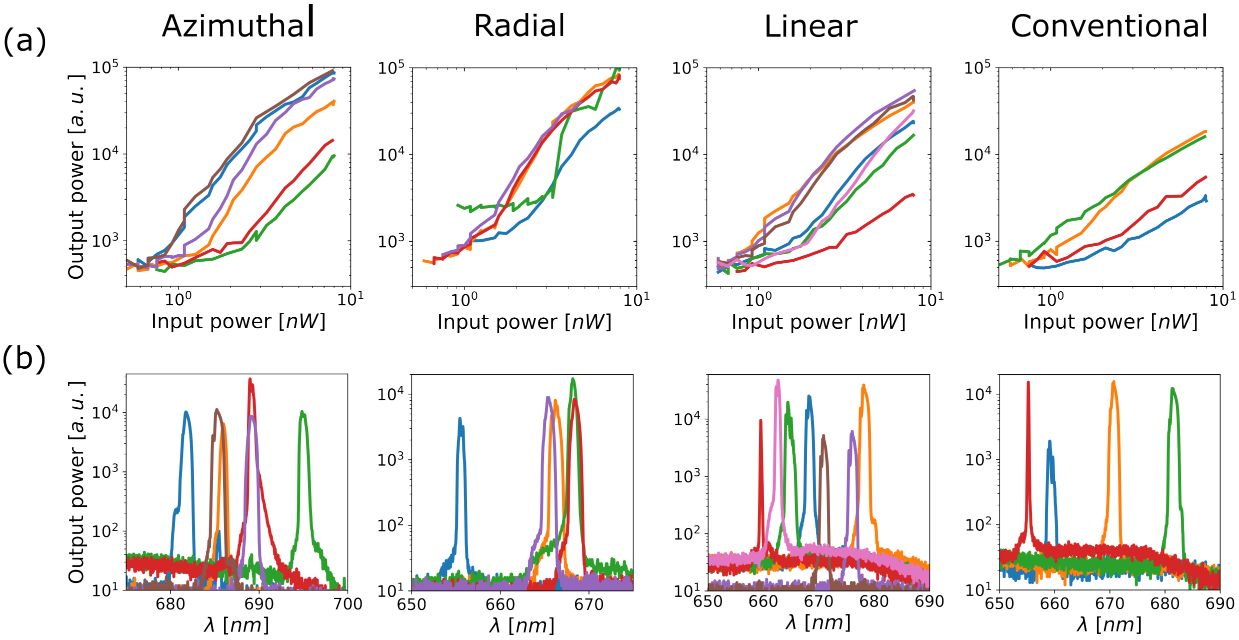


Figure S9: (a) Light in – light out (L–L) curve of the selected ensemble of nanolasers. (b) Photoluminescence spectra of nanolasers. Both type of graphs share the same color legend.

(RP), 660 nm – 678.03 nm (LP), 657 nm – 681 nm (Conv).

- Interval of lasing threshold: 1.2 nW – 2.7 nW (AP), 1.6 nW – 2.84 nW (RP), 1.07 nW – 2.24 nW (LP), 0.29 nW – 1.11 nW (Conv).

- Interval of input-output power efficiency slope: 1506 a.u./nW – 13052 a.u./nW (AP), 6390 a.u./nW – 17107 a.u./nW (RP), 514 a.u./nW – 8610 a.u./nW (LP), 403 a.u./nW – 2764 a.u./nW (Conv).

- Interval of max Q = 2223 – 3652 (AP), 2093 – 2700 (RP), 3148 – 4851 (LP), 1676 – 4773 (Conv).

- Interval of average cavity diameter = 0.929 $\mu$m – 0.952 $\mu$m (AP), 0.875 $\mu$m – 0.915 $\mu$m (RP), 0.865 $\mu$m – 0.904 $\mu$m (LP), 0.880 $\mu$m – 0.950 $\mu$m (Conv). The fabricated structures resulted in a $\sim$ 50 nm larger diameter than what they were designed for.

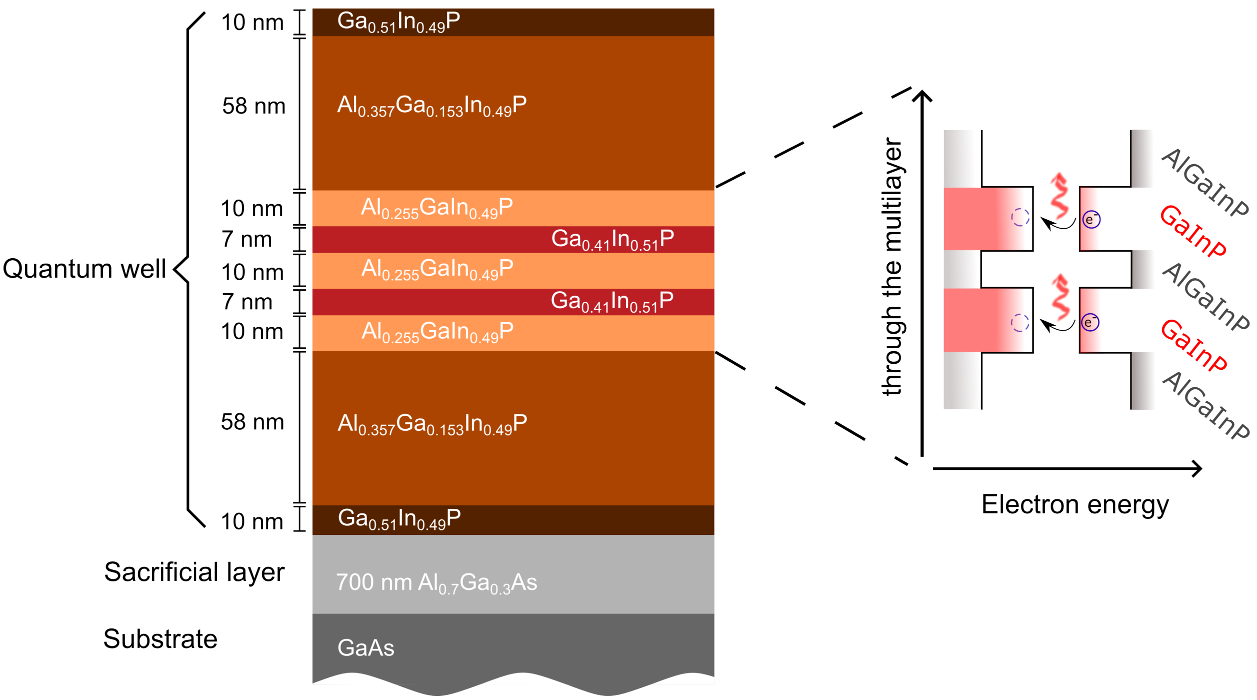


Figure S10: Composition of the whole multilayer stack that composes the double quantum well.

## S9. Full Layer Structure of the Quantum Well Wafer

The structure was grown by metalorganic vapour phase epitaxy in a low-pressure (150 Torr) reactor on (100) GaAs substrates with a misorientation angle of 10 deg towards $< 111 >$A. Trimethyl alkyls were used as precursors for the group III elements, and phosphine and arsine were used as precursors of group V elements. Hydrogen was used as carrier gas. The full layer structure of the quantum well wafer can be seen in Figure S10.

## References

(1) Lalau-Keraly, C. M.; Bhargava, S.; Miller, O. D.; Yablonovitch, E. Adjoint shape optimization applied to electromagnetic design. *Optics Express* **2013**, *21*, 21693.

(2) Miller, O. D. Photonic Design: From Fundamental Solar Cell Physics to Computational Inverse Design. Ph.D. thesis, University of California, Berkeley, 2013.

(3) Sell, D.; Yang, J.; Doshay, S.; Yang, R.; Fan, J. A. Large-Angle, Multifunctional Metagratings Based on Freeform Multimode Geometries. *Nano Letters* **2017**, *17*, 3752–3757.

(4) Mansouree, M.; McClung, A.; Samudrala, S.; and Arbabi, A., Large-Scale Parametrized Metasurface Design Using Adjoint Optimization. *ACS Photonics* **2021**, *8*, 455–463.

(5) Novotny, L., and Hecht, B., *Principles of Nano-Optics*; Cambridge University Press, 2006.

(6) Rothwell, E.J., and Cloud, M.J. , *Electromagnetics (2nd ed.)*; CRC Press, 2009.

(7) Kim, D. C.; Hermerschmidt, A.; Dyachenko, P.; Scharf, T. Inverse design and demonstration of high-performance wide-angle diffractive optical elements. *Optics Express* **2020**, *28*, 22321.

(8) Callewaert, F.; Butun, S.; Li, Z.; Aydin, K. Inverse design of an ultra-compact broadband optical diode based on asymmetric spatial mode conversion. *Scientific Reports* **2016**, *6*, 1–10.

(9) Wang, J.; Shi, Y.; Hughes, T.; Zhao, Z.; Fan, S. Adjoint-based optimization of active nanophotonic devices. *Optics Express* **2018**, *26*, 3236.
